# Socio-ecological predictors of dietary inflammatory scores and associations with childhood and adolescent adiposity: A protocol for a rapid scoping review of observational studies

**DOI:** 10.1371/journal.pone.0286200

**Published:** 2023-05-25

**Authors:** Kristina Vingrys, Helen McCarthy, Ricardo Segurado, James R. Hébert, Catherine M. Phillips

**Affiliations:** 1 School of Public Health, Physiotherapy, and Sports Science, University College Dublin, Belfield, Dublin, Ireland; 2 Institute for Health and Sport, Victoria University, Melbourne, Victoria, Australia; 3 First Year College, Victoria University, Melbourne, Victoria, Australia; 4 UCD Centre for Support and Training in Analysis and Research, University College Dublin, Belfield, Dublin, Ireland; 5 Cancer Prevention and Control Program and Department of Epidemiology and Biostatistics, Arnold School of Public Health, University of South Carolina, Columbia, United States of America; 6 Department of Nutrition, Connecting Health Innovations LLC, Columbia, United States of America; Università degli Studi di Milano, ITALY

## Abstract

**Introduction:**

Diet-related inflammation is associated with adiposity. Obesity and inflammation in early life may have adverse health outcomes in later life; however, the socio-ecological predictors of a pro-inflammatory diet in childhood and adolescence are not well understood. This rapid scoping review aims to summarise the current state of research from observational studies investigating socio-ecological predictors (childhood, parental, familial, demographic and chronobiological risk factors) and their association with diet-associated inflammation and adiposity in children and adolescents.

**Methods:**

This scoping review will be conducted using the frameworks based on the Joanna Briggs Institute and Arksey and O’Malley and the Population, Concept and Context (PCC) mnemonic. Searches were conducted in OVID Medline, Cinahl and Embase, with adaptations as required. The piloted study selection process will utilise two reviewers for study selection, with reference lists checked for included studies. A third reviewer will moderate disagreements. Data will be extracted by one reviewer and calibrated by a second reviewer.

**Results:**

The results will be reported using the Preferred Reporting Items for Systematic reviews and Meta-Analyses extension for Scoping Reviews (PRISMA-ScR) checklist and PRISMA-ScR flow diagram. The main findings will be synthesised into themes and concepts narratively. Tables and graphs will present frequencies, study details and categorical descriptions.

**Discussion:**

This scoping review will provide an overview of the research conducted to date regarding predictors of diet-related inflammation in childhood and their associations with adiposity. Better understanding of the factors associated with a more inflammatory diet in childhood may be useful for clinicians and policy makers when designing and implementing health interventions.

## Introduction

Childhood obesity has reached epidemic proportions worldwide [1–3] and has major consequences for producing short- and long-term adverse health outcomes [4–6], detracting from quality of life and creating a huge societal burden in terms of lost productivity and medical costs [7–9]. The risk factors that increase the risk of a child developing overweight, known as predictors of child overweight, are important in understanding the multifactorial aetiology of childhood overweight [10]. The interplay between multiple risk factors are recognised in adiposity research, that highlights the importance of foetal and early childhood exposures [11], foetal programming [12] and ecological predictors, including parental, familial, societal and demographic characteristics [10]. However, the relationships among predictors of diet-associated inflammation and adiposity in children, remains unclear [13].

Critical phases of childhood development represent distinct periods during which physical alterations occur that may increase the prevalence of subsequent obesity [14]. Importantly, predictors may have different effects on childhood dietary inflammation and associations with childhood adiposity at different times in childhood. For example, early adiposity rebound (AR) in childhood is a risk factor for overweight [15], and early AR has been correlated with diet and lifestyle factors including sugar-sweetened beverage consumption [16].

Chronic inflammation, with persistently elevated levels of circulating pro-inflammatory biomarkers, is characteristic of obesity [17, 18] and can be systemic or tissue-specific simmering inflammation [19]. Dietary intake is a key environmental factor that contributes to body weight outcomes, chronic inflammation and immune response [18, 20, 21].

**Fig 1:**
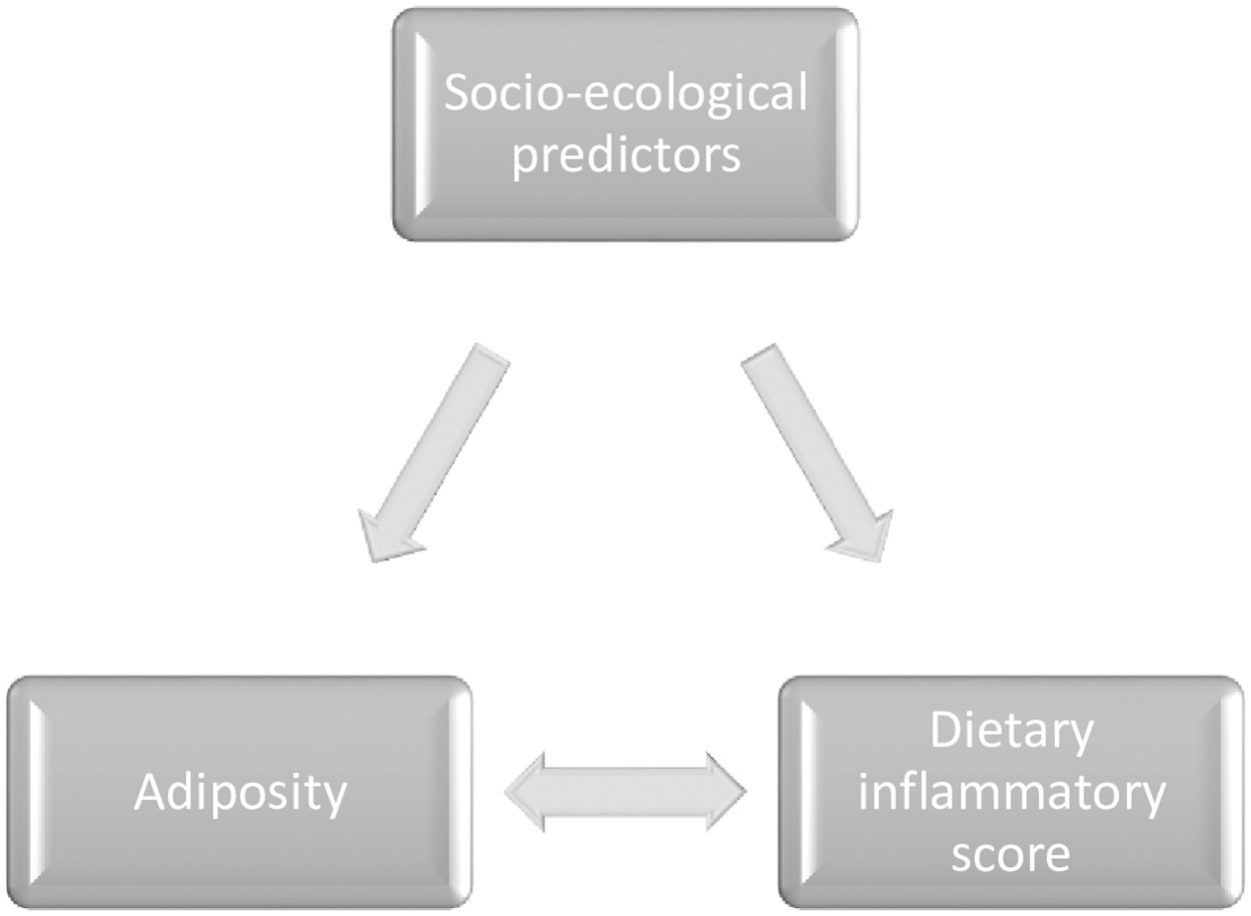
Conceptual overview of associations to be explored in the scoping review; socio-ecological predictors (including child personal, parental, familial and chronobiological factors), dietary inflammatory scores and adiposity, in childhood and adolescence.

Associations between dietary components as ecological predictors of diet-related inflammation are relatively well understood. For example, it is known that specific food components (ultra-processed foods, added sugars, saturated fatty acids) and dietary patterns (Western-style diet) are associated with elevated biological inflammatory markers in children and adolescents [22]. However, the wider socio-ecological predictors of more pro-inflammatory diets, relating to the child and familial demographic characteristics, are under-researched. Likewise, the associations between dietary inflammation and childhood adiposity are unclear (Fig 1), with overweight and obesity associated in some studies [23–26], but not others [27, 28].

When assessing diet as an environmental factor associated with inflammation in children, several indices have been used to score the inflammatory potential of the diet, including the Dietary Inflammatory Index (DII^®^) [29, 30] and variations: the energy-adjusted DII (E-DII^™^) [21]; the Children’s DII (C-DII^™^) [21, 28] and the child Dietary Inflammatory Score (cDIS) [31]. The DII and variations such as the E-DII evaluate the inflammatory potential of dietary intake by estimating the degree of inflammatory potential of specific nutrients and foods using a score on a scale ranging from around -9 to +8 [29]. Higher scores, reflecting more pro-inflammatory diets, have been associated with increased risk of obesity and non-communicable diseases in adults [21, 28, 32] a higher risk of obesity in children at ages 5 and 9 years in the Lifeways cohort [33]. A recent systematic review also found an increased risk of cardiometabolic, inflammatory and adiposity related measures associated with higher E-DII and C-DII in children and adolescents from 6 studies [34], suggesting that cohort studies are a useful model to explore socio-ecological predictors of dietary inflammation in children and their associations with adiposity, where familial, parental and personal factors may be at play.

### Aims and objectives

Prediction research is useful to help forecast future disease occurrence. This includes predictor finding studies (of risk factors) and predictor modelling studies [35, 36]. The rationale that underpins the aims of this scoping review is that studies designed to find predictors have largely focussed on associations between specific dietary components and inflammatory markers, relating to cardiometabolic factors and adiposity [31, 34]; however, the wider universe of socio-ecological predictors of childhood dietary inflammation and their associations with childhood adiposity are not well studied. Moreover, observational studies can estimate differences in risk attributed to predictors (risk factors) [37] with the concomitant potential to identify candidate predictors for prediction modelling studies. Therefore, as an exploratory analysis, establishing the extent, and types, of prediction modelling studies available will assist in understanding whether new models are needed or whether existing models can be feasibly adapted to undergo validation or updating.

Therefore, the aims of this scoping review are to:

1. Summarise the current state of research and gaps in the literature investigating the association between dietary inflammation scores and adiposity in children and adolescents in observational studies;
2. Summarise the current research reporting associations between socio-ecological predictors (risk factors), or prediction models, of dietary inflammation in childhood and adolescence, measured by dietary inflammation scores and associations with adiposity in observational studies; and
3. Describe whether there is any variation of risk at different childhood ages (early, middle, and late childhood) of a more pro-inflammatory diet associated with adiposity.

The objective of this review is to subsequently inform future research investigating predictors and prediction models of dietary inflammation and obesity in children in observational studies. The feasibility of prediction modelling from potential candidate predictors will also be explored [38].

## Methods

The scoping review methods were developed using the Joanna Briggs Institute’s (JBI) updated guidance on scoping reviews [39], incorporating extensions based on the Arksey and O’Malley framework [40–42]. The reporting of this protocol followed the PRISMA for systematic review protocols (PRISMA-P) checklist [43–45] (S1 Table). The reporting of the subsequent scoping review will follow the Preferred Reporting Items for Systematic reviews and Meta-Analyses extension for Scoping Reviews (PRISMA-ScR) checklist [45]. Within the scoping review framework, a rapid evidence synthesis approach has been adopted to accommodate completion timelines whilst maintaining methodological rigour. This includes selected methodological efficiencies identified from other rapid reviews [46, 47], including applying search limits (restriction to humans, date range, publication types and English language), and maintaining independent double reviewers for study selection; however, utilising a single reviewer for extraction with calibration by a second reviewer. Any deviations from the protocol will be agreed by the reviewers and documented [39]. The database searches were performed in July 2022.

### Stage 1. Research question identification

To formulate the key factors and criteria being investigated in this review, the Population, Concept and Context (PCC) mnemonic was used as recommended by JBI [48].

#### Population

The target population consists of children and adolescents ≥2 years and <19 years of age based on World Health Organization definitions [49, 50]. The children included in our study are restricted to ≥ 2 years of age to limit to children with dietary intake coming mostly from foods rather than breast milk. Given the different stages of growth within childhood and adolescence, the population will also be considered categorically as early, mid and late childhood and adolescence. The target population includes generally healthy children and adolescents that may have overweight or obesity. Those diagnosed with chronic diseases (including diabetes and cancer) will be excluded.

#### Concept

The breadth of inquiry will include studies with outcomes in the phenomena of interest; predictors of diet-related inflammation and adiposity. ‘Predictors’ in this study refers to risk factor variables in relation to a more pro-inflammatory diet and their associations with adiposity, including socio-ecological predictors: biological, familial or environmental. Socio-ecological predictors will be adapted from ecological factors in childhood obesity described by Davison and Birch (2001) and matched to potential cohort data and include:

Child characteristics include sex, age, adiposity measures and risk factors: sedentary behaviour, physical activity;

Parental styles and familial characteristics include parental dietary intake, weight status, activity patterns, smoking and alcohol intake;

Parental demographic characteristics include ethnicity, work hours, leisure time, socio-economic status and education level;

Chronobiological characteristics include meal timing and sleep.

Specific dietary factors (food groups and nutrients) will not be the focus as these associations with the C-DII are comparatively well explored in contrast to wider socio-ecological risk factors [32].

As an exploratory analysis, studies reporting prediction modelling of dietary-related inflammation also is of interest. These include studies developing new prediction models or validating or updating existing prediction models [51]. Concepts for prediction modelling were identified from the transparent reporting of a multivariable prediction model for individual prognosis or diagnosis (TRIPOD): The TRIPOD statement [51], using the TRIPOD Checklist for Prediction Model Development and Validation. Diet-related inflammation in this study was defined as biological inflammation related to dietary components that are measured by indices or scores including the DII and variations including the E-DII, C-DII and cDIS. Adiposity will be defined as the amount of body fat [52] and measured by proxy outcomes including body weight, body mass index (BMI), abdominal circumference, skinfold thickness and weight-for-height. Studies of human subjects with overweight or obesity as indicators of adiposity will be included provided that subjects were otherwise generally healthy.

#### Context

The context of this scoping review is in observational studies, defined as studies with either cohort, case-control or cross-sectional designs [53] that will have been reported in peer-reviewed journal publications, articles in press that are published online and reviews or conference papers, from 2009 to the time of running the searches. The earliest date limitation reflects 2009, the year when the concept of the DII was first published [30]. Peer-reviewed literature is prioritised in this context to align with the rapid methodology and recognising that peer-review is as an important process aimed at ensuring study quality [54]. The research questions will be reviewed as literature is obtained. The primary and secondary research questions identified were:

### Primary questions

1. What is known from the current published literature in observational studies about the association between a more inflammatory diet, measured by higher dietary inflammation scores, and association with adiposity in childhood and adolescence?
2. What is known from the current published literature in observational studies about socio-ecological predictors of a more inflammatory diet, measured by higher dietary inflammation scores, and their association with childhood and adolescent adiposity?

### Secondary questions

3. What is known from the current published literature about the variation in risk of a more inflammatory diet at different stages of childhood and adolescence (early, middle and late) identified from the primary research questions?
4. What is known from the current published literature about the variation in risk of a more inflammatory diet and association with chronobiological factors (such as meal timing and sleep)?
5. What is known from the current published literature about prediction modelling developed in observational studies, investigating candidate predictors in determining the risk of dietary inflammation in childhood and adolescence?

### Stage 2. Identifying relevant studies—Search strategy

The search strategy was developed primarily by one reviewer with input from the research team and a research librarian. Variations were discussed and agreed by the reviewers and were documented. As the search process is recommended to be iterative, there is provision to allow modification of keywords, search terms and information sources as the reviewers become increasingly familiar with the literature [39, 40].

The databases to be searched were OVID Medline, Cinahl and Embase. Initial scoping searches were piloted in OVID Medline and adapted as required for other databases. The search results were documented and the full search strategy of one major database (OVID Medline) is provided.

Where databases allowed, limits were used to restrict studies to ‘human’, ‘title and abstract’ though this was trialled in each database to determine impact on the output specificity and sensitivity. The search strategy was piloted and refined before final run and modified minimally as required for each database. The search strategy was finalised after trialling using a combination of key words and phrases, synonyms and controlled vocabulary (e.g., MeSH) (Table 1).

**Table 1:** Electronic search strategy for the scoping review to be adapted for each database.

SEARCH NO.	SEARCH STRINGS
**POPULATION**	
*1*	(child* or pediatric* or paediatric* or toddler or adolescent* or youth or teenager* or teen* or tween* or young adult*)
**CONCEPT**	
*2*	(dietary inflammatory index or DII or C-DII or cDIS or E-DII or dietary inflammation score or dietary inflammatory score or dietary inflammation potential or anti-inflammatory diet or anti-inflammatory diet or pro inflammatory diet or pro-inflammatory diet or inflammatory diet or inflammatory potential or diet-related inflammation)
*3*	(obes* or adipos* or overweight or skinfold* or waist to hip ratio or waist-to-hip ratio or waist-hip ratio or WHR or fat mass or bioelectrical impedance or DEXA or overnutrition or growth or growth trajectory or waist circumference or body mass index or BMI or abdominal circumference or weight-for-height or weight for height or cardiometabolic or metabolic)
*4*	2 and 3
*5*	((parent* or mother* or father* or maternal or paternal or famil* or sibling* or brother* or sister* or grandparent* or guardian* or carer* or care giver or caregiver or demographic or income or low-income or low income or education or socio-economic or socioeconomic or socio economic or ethnic* or race or racial or cultur* or sex or gender or physical activity or sedentary or lifestyle or smoking or sleep or sleep-wake or wake or television or screen time or alcohol or age or provider or breastfeeding or breast feeding or solids or meal or meal tim* or timing or breakfast or lunch or dinner or snack* or meal skipping or fasting or diet* intake or food intake or energy intake or meal pattern or diet* pattern or chronobiology or ((predict* or diagnostic or prognostic) and (model or study))) and (characteristics or risk factors or risk or association or correlation))
*6*	1 and 4 and 5
**CONTEXT**	
*7*	limit 6 to (English language and Humans and Year 2009-Current)

### Stage 3. Study selection

The inclusion and exclusion criteria for the searches are matched to the PCC (Table 2). These criteria may be changed *post-hoc* as familiarity with the literature increases [40] and on discussion with the research team. The study selection process will be piloted by the reviewers before the formal run [39]. Title and Abstracts of the identified studies will be screened by two reviewers based on the inclusion and exclusion criteria. Reasons for exclusion will be recorded. Following abstract review, the first round of selected studies will proceed to full text screening for final selection by two reviewers. The reference lists of included studies will be hand-searched for further studies. Disagreements will be moderated by a third reviewer. Experts in the field will be consulted to identify other studies.

**Table 2:** Inclusion and exclusion criteria for the scoping review searches.

	Inclusion	Exclusion
**Population**	Humans Children and adolescents (≥ 2 years, < 19 years of age) Male or female Generally healthy participants that can include overweight or obesity	Children < 2 years Adults (≥ 19 years of age) Disease (e.g., diabetes, cancer)
**Concept**	Outcome reporting predictors of diet-related inflammation and adiposity, where: Predictors associated with dietary-related inflammation, including biological, familial and environmental predictors; Diet-related inflammation measured by scores e.g., DII, E-DII, C-DII, DII, cDIS Adiposity measured by body weight, BMI, WHR, DEXA, bioelectrical impedance, fat mass, abdominal circumference, skinfold thickness, weight-for-height; Prediction modelling studies of dietary-related inflammation	Supplements or medication interventions Complex lifestyle interventions Unreliable assessments of outcome measures
**Context**	Observational studies Quantitative Where more than one study has been published from the same cohort, the most comprehensive study will be selected (e.g., the longest follow-up or entire cohort or most detail will be retained)	Qualitative studies *in-vitro* studies Animal studies Duplicated data Irrelevant or incomplete data
**Publication type**	Full text peer-reviewed journal publications Articles in press published online Reviews Conference papers	Articles where full text is not obtainable. Editorials, case-series and case reports without comparators, theses, books and book chapters Non-peer reviewed publications
**Year range**	January 2009 to present	Prior to January 2009
**Language**	English language	Articles not in English language

The search outputs will be combined within Endnote (version 20.3, Clarivate Analytics 2020). The citations will be uploaded to Covidence systematic review software [55] for screening and data extraction and duplications removed. Online training videos are available to support the review team. The process and results of the searches will be documented in a shared file including search dates, key terms used and number of results and translated to results in a flow diagram. If sources are excluded at the full-text review stage, details will be attached to the review as an appendix [39]. Study records will be maintained on University drives with password protection for online files.

Critical appraisal of the selected studies will not be conducted as it is not required to meet the specific aims of this scoping review [39].

### Stage 4. Data extraction (charting the data)

Extracted information will be synthesised using a piloted electronic data extraction table in Microsoft Excel, using the first 4 studies retrieved [56] and revising the extraction form as needed [57]. One reviewer will extract the data and a second experienced reviewer will verify the extracted data for calibration [57, 58].

Data underlying the findings will be made available at the time of publication as part of the manuscript or included as supplemental material. Data will be presented in tables/charts, supported by a narrative summary discussing the findings in relation to the objectives of the review.

The final studies selected will undergo syntheses into themes to extract and record the relevant findings for thematic arrangement into outcome domains and comparative overview. Categorisation into the outcome domains and themes will utilise an iterative approach to guide conceptualisation of the thematic structure, undertaken by one reviewer and then referred to the review team for consensus [56]. These may include various sub-headings identified under modifiable and non-modifiable predictors as previously by Chi and colleagues [59].

If authors need to be contacted for clarification or to request additional data, it will be documented and reported in the review.

The following data will be extracted:

Study details: Author(s), publication year, study location.

Study methodology: Aims, study type, methodology (eligibility criteria for participants), comparator, duration, study population/setting (e.g., general population, primary care, number and location of centres), outcome measures. Additionally, for prediction modelling studies this information will be recorded: key study dates (start and end of accrual), candidate predictors and how/when they were measured, model development methodology, model performance, discrimination, calibration, validation, sensitivity, specificity, predictive capacity.

Results:

Number of participants/sample size, participant characteristics (demographics, age, sex, clinical), missing data, mean follow up/length of investigation, number of outcome events, loss to follow up, predictors/risk factors identified.

Additionally, for predictor finding studies hazard ratios (HR), risk ratios (RR) or odds ratios (OR) from the most fully adjusted model for the highest versus the lowest exposure categories or levels and corresponding 95% Confidence Intervals (CIs) for exposure categories, or P-values, and authors’ conclusions will be reported.

Additionally, for prediction modelling studies: type of study (development or validation or updating), number of participants/sample size, final model predictors, goodness of fit test for models, probabilities (ORs, AUROC), sensitivity, specificity, accuracy, RMSE, prediction intervals, R^2^ values, unadjusted associations between candidate predictors and outcome, prediction model performance measures (with CIs), and proposed use of the prediction model will be reported.

## Results

### Stage 5. Collating, summarizing and reporting results

To ensure accurate and transparent reporting, each stage of the review will be reported using the Preferred Reporting Items for Systematic reviews and Meta-Analyses extension for Scoping Reviews (PRISMA-ScR) checklist [45]. A narrative synthesis will be conducted for the main findings.

First, the search results will be presented narratively and in a PRISMA-ScR flow diagram to present an overview of the identification, screening, assessment of eligibility and selection processes [39]. The main results will be summarised to include an overview of the themes and concepts categorised in outcome domains and types of evidence retrieved. Descriptive results will be narratively reported and visually presented in tables and graphs to report frequencies such as counts of concepts, study locations, categorical description of outcome measures [39]. The results will be contextualised with the review aims and objectives as well as consider relevance to population groups, limitations, including publication bias, and future directions. The protocol and ensuing review will be submitted for publication in a peer-reviewed journal.

## Discussion

Discussion of the results of the ensuing rapid scoping review will be guided by the PRISMA ScR checklist and will include a summary of evidence as described in Stage 5, a discussion of the limitations and strengths of the review, and concluding remarks to interpret the results, implications and future directions.

Understanding the predictors of childhood dietary-related inflammation and its association with childhood obesity is important in the development of public health prevention strategies to reduce the burden that obesity places on individuals and society, including health trajectories and outcomes into adolescence and adulthood [60]. This review will add to our understanding of the complex factors that contribute to the development of diet-related inflammation in childhood that is emerging as a serious co-morbidity associated with childhood obesity but is not yet well understood.

## Supporting information

S1 Table PRISMA for systematic review protocols (PRISMA-P) checklist. (PDF)
