# Supplementary material for: Socio-ecological predictors of dietary inflammatory scores and associations with childhood and adolescent adiposity: A protocol for a rapid scoping review of observational studies
Source: PLoS One. 2023 May 25;18(5):e0286200. doi: 10.1371/journal.pone.0286200 (PMC10212097; doi:10.1371/journal.pone.0286200)
Supplement: S1 Table — (PDF) [file pone.0286200.s001.pdf]

S1 Table. PRISMA for systematic review protocols (PRISMA-P) checklist

This checklist has been adapted for use with protocol submissions to Systematic Reviews from Table 3 in Moher D et al: Preferred reporting items for systematic review and meta-analysis protocols (PRISMA-P) 2015 statement. Systematic Reviews 2015 4:1 [1, 2].

Section/topic	#	Checklist item	Information reported		Section reported
			Yes	No	
**ADMINISTRATIVE INFORMATION**					
**Title**					
Identification	1a	Identify the report as a protocol of a systematic review / scoping review	☒	☐	Title
Update	1b	If the protocol is for an update of a previous systematic review / scoping review, identify as such	☐	☒	N/A
**Registration**	2	If registered, provide the name of the registry (e.g., PROSPERO) and registration number in the Abstract	☐	☒	N/A
**Authors**					
Contact	3a	Provide name, institutional affiliation, and e-mail address of all protocol authors*; provide physical mailing address of corresponding author. *Email address of corresponding author provided as per journal template.	☒	☐	Author information
Contributions	3b	Describe contributions of protocol authors and identify the guarantor of the review	☒	☐	Contributions
**Amendments**	4	If the protocol represents an amendment of a previously completed or published protocol, identify as such and list changes; otherwise, state plan for documenting important protocol amendments	☒	☐	Methods, paragraph 1 and Stage 3 Study selection
**Support**					
Sources	5a	Indicate sources of financial or other support for the review	☒	☐	Funding statement
Sponsor	5b	Provide name for the review funder and/or sponsor	☒	☐	Funding statement
Role of sponsor/funder	5c	Describe roles of funder(s), sponsor(s), and/or institution(s), if any, in developing the protocol	☒	☐	Funding statement
**INTRODUCTION**					
**Rationale**	6	Describe the rationale for the review in the context of what is already known	☒	☐	Aims and objectives
**Objectives**	7	Provide an explicit statement of the question(s) the review will address with reference to participants, interventions, comparators, and outcomes (PICO). Note:	☒	☐	Stage 1 Research question

Section/topic	#	Checklist item	Information reported		Section reported
			Yes	No	
		Population, Concept and Context (PCC) mnemonic was used as recommended by JBI [3].			identification and Table 1
**METHODS**					
**Eligibility criteria**	8	Specify the study characteristics (e.g., PICO, study design, setting, time frame) and report characteristics (e.g., years considered, language, publication status) to be used as criteria for eligibility for the review	☒	☐	Table 2
**Information sources**	9	Describe all intended information sources (e.g., electronic databases, contact with study authors, trial registers, or other grey literature sources) with planned dates of coverage	☒	☐	Stage 2 Identifying relevant studies – search strategy and Table 2
**Search strategy**	10	Present draft of search strategy to be used for at least one electronic database, including planned limits, such that it could be repeated	☒	☐	Table 1
STUDY RECORDS					
Data management	11a	Describe the mechanism(s) that will be used to manage records and data throughout the review	☒	☐	Methods, paragraph 1, Stage 3 Study selection and Stage 5 Collating, summarizing and reporting results
Selection process	11b	State the process that will be used for selecting studies (e.g., two independent reviewers) through each phase of the review (i.e., screening, eligibility, and inclusion in meta-analysis)	☒	☐	Stage 3 Study selection
Data collection process	11c	Describe planned method of extracting data from reports (e.g., piloting forms, done independently, in duplicate), any processes for obtaining and confirming data from investigators	☒	☐	Stage 4 Data extraction (charting the data)
**Data items**	12	List and define all variables for which data will be sought (e.g., PICO items, funding sources), any pre-planned data assumptions and simplifications	☒	☐	Stage 4 Data extraction (charting the data)
**Outcomes and prioritization**	13	List and define all outcomes for which data will be sought, including prioritization of main and additional outcomes, with rationale	☒	☐	Stage 4 Data extraction (charting the data)

Section/topic	#	Checklist item	Information reported		Section reported
			Yes	No	
**Risk of bias in individual studies**	14	Describe anticipated methods for assessing risk of bias of individual studies, including whether this will be done at the outcome or study level, or both; state how this information will be used in data synthesis	☒	☐	'summary of evidence', Stage 5 Collating, summarizing and reporting results
DATA					
**Synthesis**	15a	Describe criteria under which study data will be quantitatively synthesized	☒	☐	Stage 5 Collating, summarizing and reporting results
	15b	If data are appropriate for quantitative synthesis, describe planned summary measures, methods of handling data, and methods of combining data from studies, including any planned exploration of consistency (e.g., I^2, Kendall's tau)	☒	☐	Stage 5 Collating, summarizing and reporting results
	15c	Describe any proposed additional analyses (e.g., sensitivity or subgroup analyses, meta-regression)	☐	☒	
	15d	If quantitative synthesis is not appropriate, describe the type of summary planned	☒	☐	Stage 5 Collating, summarizing and reporting results
**Meta-bias(es)**	16	Specify any planned assessment of meta-bias(es) (e.g., publication bias across studies, selective reporting within studies)	☒	☐	Stage 5 Collating, summarizing and reporting results
**Confidence in cumulative evidence**	17	Describe how the strength of the body of evidence will be assessed (e.g., GRADE)	☒	☐	Stage 5 Collating, summarizing and reporting results

Note: Adaptations for scoping reviews are highlighted in red font.

REFERENCES

1. Moher D, Shamseer L, Clarke M, Ghersi D, Liberati A, Petticrew M, et al. Preferred reporting items for systematic review and meta-analysis protocols (PRISMA-P) 2015 statement. Syst Rev. 2015;4(1):1. Epub 2015/01/03. doi: 10.1186/2046-4053-4-1. PubMed PMID: 25554246; PubMed Central PMCID: PMC4320440.
2. Shamseer L, Moher D, Clarke M, Ghersi D, Liberati A, Petticrew M, et al. Preferred reporting items for systematic review and meta-analysis protocols (PRISMA-P) 2015: elaboration and explanation. BMJ. 2015;349:g7647. Epub 2015/01/04. doi: 10.1136/bmj.g7647. PubMed PMID: 25555855.
3. The Joanna Briggs Institute. Joanna Briggs Institute Reviewers' Manual: 2015 edition/supplement. South Australia: The University of Adelaide, 2015.
